# Strengthening systems of accountability for women’s leadership in the health sector

**DOI:** 10.1136/bmj-2023-078960

**Published:** 2024-07-17

**Authors:** Kent Buse, Harvy Joy Liwanag, Aaron Koay, Sapna Kedia, Sylvia Kiwuwa-Muyingo, Soon-Young Yoon, Sarah Hawkes

**Affiliations:** 1 Global Health 50/50, Cambridge, UK; 2 Healthier Societies Program, George Institute for Global Health, Imperial College London, London, UK; 3 Institute of Social and Preventive Medicine, University of Bern, Bern, Switzerland; 4 Institute for Global Health, University College London, London, UK; 5 International Center for Research on Alliance of Women, New Delhi, India; 6 African Population and Health Research Center, Nairobi, Kenya; 7 International Alliance of Women, New York, USA

## Abstract

Accountability can improve equal opportunities for women’s career progression and it must be strengthened in the health sector, argue **Kent Buse and colleagues**

People working in health sectors are generally familiar with the concept of accountability for standards and quality in delivery of care.^1^ This means that individuals and organisations are compelled to take responsibility for their actions and inactions. Mechanisms for accountability can include clinical audits, professional training requirements, and ultimately public scrutiny under the auspices of public inquiries, courts, or parliamentary review.^2^ In this analysis, we move beyond the question of accountability for healthcare delivery and explore accountability for equality of opportunity in health sector careers, with a focus on women’s leadership. We use a broad definition of the health sector to include public and private health organisations beyond those directly delivering care. 

At the top of the sector’s leadership pyramids, large inequities exist in the demographic profiles of leaders. Just 44 women were among 194 ministers of health in 2020^3^; a mere 10.4% of US Fortune 500 healthcare companies had a female chief executive in 2023^4^; and only 17 (<1%) of 2014 board members across 146 global organisations active in health are women from low income countries.^5^ These inequities persist despite multiple commitments and much advocacy for health workforces to be more equitable.^6^ For example, target 5.5 in the UN sustainable development goals (SDGs) commits 193 signatory countries to “Ensuring women’s full and effective participation and equal opportunities for leadership at all levels of decision-making.” The political declarations of the high level meetings on universal health coverage^7^ and that of the 25th anniversary of the fourth world conference on women,^8^ are among many other commitments to increasing women’s leadership.

Our analysis, part of a BMJ collection on gender equality in the health workforce,^9^ ^10^ ^11^ ^12^ focuses on India and Kenya and uses a rights based framework to assess accountability (box 1). We chose these countries because they are of particular interest to the funder of our commissioned research, but the performance of and insights from India and Kenya have lessons for other settings wishing to improve accountability for women’s leadership. Our examples highlight how accountability has been used to promote equal opportunities for women’s careers and how it could be used to further their progress to formal leadership, including in the health sector.

Box 1 **Principles of accountability^13^**

- An agency is responsible for reporting performance information in the public domain (ie, transparency)
- Progress towards targets is independently reviewed
- Remedial actions enforced in cases of failure to meet obligations

## International agreements as paths to state accountability

Several international mechanisms are available to drive accountability for women’s leadership such as the International Labour Organisation (ILO) tribunals, and the United Nations (UN) human rights special rapporteurs. However, the principal international treaty mechanism concerning women’s rights is the Convention on the Elimination of All Forms of Discrimination Against Women (CEDAW).^14^ It was adopted by the UN General Assembly in 1979 and ratified by 189 of the 193 member states.^15^ State parties to CEDAW have committed to enshrine gender equality in their domestic laws and implement special measures to accelerate substantive equality in the workplace including legislation governing equal pay, workplace protections, pensions, flexible working and work-life balance, among other domains.

State parties are required to report every four years to the independent CEDAW committee of experts, which evaluates countries’ progress in upholding their treaty obligations.^16^ The latest available reports from India (2012-13) and Kenya (2016) show that both have assigned responsibility to an agency (National Commission for Women in India, Gender and Equalities Commission in Kenya) to report information and go through a process of independent review. The countries therefore meet two of the three principles of accountability (box 1). 

CEDAW’s final recommendations in response to state reports include concerns raised in shadow reports submitted by civil society organisations (CSOs). Such submissions could therefore help heighten awareness of women’s leadership. Shadow reports have previously been used to highlight women’s concerns in tobacco control,^17^ and CSO advocacy campaigns have supported the adoption of CEDAW principles in the development of local government ordinances and policies.^18^ At least 12 Kenyan CSOs submitted shadow reports in 2016, which could help bolster the accountability of the government. No shadow reports were submitted during India’s last review in 2012-13.

Both countries address CEDAW obligations through a range of domestic legislation, including enshrinement of gender equality in their constitutions and the adoption of legal efforts that set a one third quota for women’s representation in political leadership and government service. Challenges, however, remain. The CEDAW committee has highlighted problems such as the absence of a comprehensive national law in India that localises its international obligations, and the discretion of Kenya’s parliament not to implement CEDAW provisions that contradict local customs and beliefs.^19^ In both countries the absence of remedial action exposes the limits of the CEDAW process, which is vague on the extent it is able to compel governments to act.^20^ For example, India has failed to respond to issues raised by the CEDAW committee in its last report despite two reminder letters from the CEDAW rapporteur.^21^

### Accountability through sustainable development goal reviews 

Another mechanism for increasing women’s leadership is the high level political forum (HLPF) on sustainable development, which reviews progress towards the SDGs.^22^ Integrated into the HLPF are voluntary national reviews, which are meant to be country led, transparent, participatory, people centred, and with a focus on people left furthest behind.^23^ Unlike CEDAW, participation in the HLPF is voluntary, and responsibility for reporting does not necessarily lie with an authority that holds a specific mandate for gender equality.

The voluntary national reviews synthesis report for 2022^24^ reviewed progress towards SDG target 5.5 (women’s full and effective participation and equal opportunities for leadership) in 44 countries but did not include India and Kenya, which each reported in both 2017 and 2020. While progress was reported in some areas (eg, in political leadership in 10 countries), the synthesis report did not explicitly describe progress on the proportion of women in managerial positions (indicator 5.5.2). Only 86 countries have reported on this indicator since 2015, and neither India nor Kenya have reported.^25^ As with CEDAW, the HLPF system is transparent but not independent. Furthermore, the HLPF process is vague about the consequences of states not submitting reports, and there do not seem to be any penalties for states not reporting or failing to make progress on these targets.

## Organisational accountability for women’s career progression

State led measures to respect, protect, and fulfil rights to gender equality, including in the workplace, are fundamental to human rights, including that of non-discrimination. In addition, employers can have critical roles in creating conditions for career equality and supporting women’s leadership, particularly when employer actions specifically acknowledge and address their underlying “inequality regimes.”^26^ Other articles in this BMJ collection identify the multiple levels across societies that drive workplace inequalities,^11^ ^12^ and the importance of more targeted policy interventions at the level of organisations and employers to promote career equality.^9^ Our research in India and Kenya finds that employment inequalities are reinforced through entrenched patriarchal norms that persist in many health workplaces and which perpetuate gendered occupational segregation, devalue professions that predominantly comprise women (eg, nursing), and embed biases and discrimination that perceive men (not women) as leaders.^12^

More globally, a systematic review of interventions to advance women in healthcare leadership described five categories of effective actions that organisations can take to promote equality: organisational processes (eg, supporting flexible working); awareness and engagement (eg, increasing male allyship); mentoring and networking (eg, peer support for women); leadership development (eg, encouraging women to apply for leadership roles); and tools aiming to reduce inequalities in recruitment, retention, and promotion and for measurement and evaluation (eg, evaluation of organisational culture).^27^ The authors of the systematic review note that “leadership commitment and accountability were critical in championing these policies and practices,” although the exact mechanisms of accountability were not detailed.^27^ Various approaches can be taken to monitor organisational progress and ensure remedial action when required (box 1).

### Monitoring

Five broad monitoring approaches could be expanded to encompass performance on efforts leading to gender equality in the workplace. The first concerns scrutiny of organisational performance. For example, in fulfilling its diversity, equality, inclusion, and belonging policy, Population Services International routinely assesses the gender diversity of its leadership and shares the data with its employees and board.^28^ Second, annual reporting of environment, sustainability, and governance (ESG) by many private sector companies includes a focus on gender diversity, inclusion, and pay equity among executive officers and corporate boards. Data from 2023 found that 99% of US companies in the Standard and Poor 500 index (including healthcare sector companies) report ESG, and 40% report on diversity and inclusion.^29^ Efforts are underway to make such reporting mandatory in several jurisdictions.^30^

Third, external accreditation for progress towards gender equality occurs through charter mark type initiatives that cover issues related to workplace culture and practice. For example, the Athena Swan Charter promotes gender equality in higher education and research careers in the UK.^31^ Such charter marks serve to monitor progress towards specific equality goals and also apply a level of conditionality in resource access; expanding this approach to the health sector could be considered. Fourth, independent monitoring related to gender equality within organisations is provided by organisations such as ours, Global Health 50/50. Although lacking the power to enforce remedial action, we aim with this independent mechanism to encourage organisational change towards fairer working practices, which we have documented in numerous organisations.^32^ Fifth, in many workplaces, monitoring and advocacy by labour unions or staff associations could serve to enhance accountability for equality in leadership. For example, the UNAIDS Staff Association has a working group that seeks to hold the organisation accountable for implementing its gender action plan.^33^

### Remedial action

Several mechanisms oriented towards remedial action have been used successfully to encourage accountability, including litigation and collective action. For instance, in 2005, around 1500 women workers in the UK’s National Health Service won an employment tribunal on the grounds of pay discrimination.^34^ Broader collective action can also drive corporate action on gender equality in the workplace. In 2023, the Africa Women Journalism Project launched a campaign challenging Kenyan companies to publish their gender pay gaps to accelerate progress on the country’s equal pay law.^35^

## Towards stronger systems of global and organisational accountability

The health sector, characterised by a female dominant workforce but with men dominating leadership, has not delivered on either legally mandated or voluntary commitments to equality of career opportunity including in leadership. Change is occurring,^36^ but slowly. Establishing and strengthening accountability mechanisms offers an additional route towards hastening change.

Monitoring in international and organisational systems of accountability is currently piecemeal, resulting in incomplete pictures of whether and what progress is being made. This could be improved, for example, by mandating states to report national progress (or lack thereof) in global accountability mechanisms. There is also a deficit of clear avenues for remedial action. Understanding what works to strengthen systems of accountability at global and organisational levels is currently limited by a relative absence of evidence and more systematic evaluation is needed.

The potential of using CEDAW or HLPF mechanisms to hold employers, including those in the health sector, to account for their commitments to equality in women’s leadership is not being realised. This may be the result of lack of awareness among health sector stakeholders of these mechanisms, a deficit of gender disaggregated workforce, and leadership data, and the absence of authoritative remedial actions. For CEDAW, stakeholders in the health sector could use three routes to enhance equitable leadership: advocating that state reporting to CEDAW includes provisions that explicitly support women’s equal leadership, including disaggregated by sector; encouraging CSOs to ensure women’s leadership is included in shadow reports; and making use of the optional protocol,^37^ which provides a mechanism for the CEDAW committee to hear individual complaints about violations of the treaty. The optional protocol has so far been used to investigate 11 cases against countries on domestic violence, parental leave, and forced sterilisation.^38^ The protocol could also serve to sanction countries where there is systematic discrimination against women in assuming leadership roles.

HLPF reporting could be enhanced by ensuring countries meet their obligations to collect and report data on the proportion of women in managerial positions, including disaggregated by sector (eg, health, education, finance, law, etc). These data should be included in the synthesis reports of voluntary national reviews, together with relevant findings from CEDAW reports. Health sector organisations should also engage with CSOs that produce independent reports on SDG progress to include issues related to workplace equality in their reports. 

Collective action, including through litigation at the organisational level, is a powerful lever, but it is an expensive and time consuming route for many employees. Holding health sector employers to account through a performance related charter mark system may be a more feasible and affordable alternative for system-wide change, and a charter mark should be developed for health sector workforces globally.

Commitments for a more equitable workplaces are little more than empty rhetorical statements without systems of accountability. The struggle to facilitate women’s leadership needs disruption. More systematically strengthening systems of accountability can provide that disruption. But we should not overlook the argument that equality in career opportunities (including leadership) is a starting point.^10^ What we also need are the types of leaders who will deliver system-wide transformational change—in other words, efforts to promote a model of feminist leadership that eliminates systemic and overlapping inequalities among all people working in health sectors and promotes social justice.

**Key messages**

- Most governments and many organisations working in the health sector have committed to gender equality through numerous international agreements and calls to action
- Nevertheless progress falls short of substantive equality in leadership opportunities for women
- Laws and organisational policies represent potentially critical levers for change but are ineffective without accountability mechanisms to ensure their implementation
- Monitoring of progress is largely voluntary and lacks independence while mechanisms to ensure remedial action are lacking
- Mandatory reporting on women’s representation and a global charter mark system are among several actions we identify which could help hold countries and health sector organisations to account for gender equality

## Notes

Contributors and sources: This article builds on two decades of research and practice in global governance for health, including on soft law and accountability by KB and on research and practice by SH and KB on gender and health, including over the past seven years as co-founders and co-chief executives of Global Health 50/50, a research-cum-advocacy, not-for-profit organisation that seeks to enhance accountability for gender equality in health. KB is a former staff member of UNAIDS, and member of its staff association. HJL, AK, and SYS contributed insights to article development, drawing on their expertise and experience in health systems research in low and middle income countries in Asia (HJL), gender and health research (AK) and advocacy and engagement for women’s rights in the United Nations system (SYS). KB and SH led the framing of the analysis and drafting of the article with HJL and AK co-writing different sections. SK, SKM, and SYS advised and reviewed the draft and revisions. All authors approve the final submission. KB is the guarantor.

Provenance and peer review: Commissioned; externally peer reviewed.

This article is part of the BMJ collection on gender equality in the health workforce, developed in partnership with Global Health 50/50, Africa Population and Health Research Centre, and International Center for Research on Women, and funded by the Bill & Melinda Gates Foundation (INV-031372). *The BMJ* commissioned, peer reviewed, edited, and made the decision to publish these articles. The lead editors were Seye Abimbola, Jocalyn Clark, and Emma Veitch for *BMJ Global Health* and *The BMJ*.
